# Acute paraplegia caused by Schistosoma mansoni

**Published:** 2014-10

**Authors:** Mahmood D. Al-Mendalawi

**Affiliations:** *Department of Pediatrics, Al-Kindy College of Medicine, Baghdad University, Baghdad, Iraq*

***To the Editor***

With reference to the interesting paper by Algahtani et al,^1^ schistosomiasis is included among many parasitic CNS infections in immunocompromised hosts due to therapy after transplantation, or associated with human immunodeficiency virus (HIV).^2^ The Kingdom of Saudi Arabia (KSA) is among developing countries with a remarkable impact of schistosomiasis where successful schistosomiasis control programs have been implemented to eliminate this debilitating disease.^3^ However, whilst the numbers of reported HIV cases have stabilized since 2006, HIV/AIDS remains an important public health problem in KSA, both in migrants and Saudi nationals.^4^ In many regions of the world, both schistosomiasis, and HIV/AIDS are endemic, resulting in patients harboring co-infections. Because interaction with host CD4+ T cells is a characteristic of schistosome as well as HIV-1 infections, bi-directional disease effects may be sufficiently different from sequelae caused by either infectious agent alone to warrant alteration of public health approaches in areas of co-endemnicity.^5^ Studies have shown that in areas where both diseases are endemic, schistosome infections lead to increased susceptibility to infection with HIV-1, a more rapid progression to disease through more vigorous viral replication and immunosuppression, and a higher likelihood of transmitting the infection to others through both vertical and horizontal routes.^5^,^6^ Algahtani et al^1^ did well in excluding concomitant HIV in their 2 studied patients with schistosomiasis. Proper treatment of schistosomiasis in populations with increased prevalence or risk of HIV infection not only reduces schistosomiasis burden, but it also might be an effective means of limiting the transmission of HIV infections.

***Reply from the Author***

No reply was received from the author
